# Psychometric properties of the Portuguese version of the Personality Assessment Inventory

**DOI:** 10.3389/fpsyg.2025.1517623

**Published:** 2025-04-01

**Authors:** Mauro Paulino, Mariana Moniz, Octávio Moura, Daniel Rijo, Leslie C. Morey, Mário R. Simões

**Affiliations:** ^1^Center for Research in Neuropsychology and Cognitive and Behavioral Intervention, University of Coimbra, Coimbra, Portugal; ^2^Faculty of Health Sciences, Universidade Europeia, Lisbon, Portugal; ^3^Psychological Assessment and Psychometrics Laboratory (PsyAssessmentLab), Coimbra, Portugal; ^4^Faculty of Psychology and Educational Sciences, University of Coimbra, Coimbra, Portugal; ^5^College of Arts & Sciences, Texas A&M University Central Texas, Killeen, TX, United States

**Keywords:** Personality Assessment Inventory, confirmatory factor analysis, principal component analysis, Portuguese version, psychometric properties

## Abstract

**Introduction:**

Although several factor analytic studies have investigated the factor structure of the Personality Assessment Inventory (PAI), no consensus has been reached regarding the best factor solution, either considering the 11 clinical scales or the 22 full scales. Whereas some studies have found that a two-factor solution for the clinical scales and a four-factor solution for the 22 scales were the most parsimonious factor structures, other studies suggested different factor models (e.g., a three-factor solution for the 11 clinical scales, and a three- or five-factor solution for the 22 full scales). Some reasons may explain the different factor structures found in the literature, namely sample characteristics (e.g., community vs. clinical samples) and methodology (e.g., number of scales included in the factor analysis, validity criteria, factor analysis techniques, and estimators).

**Methods:**

The present study aimed to investigate the factor structure of the Portuguese version of the PAI in a sample of 900 participants (aged 18–74 years, 57.7% female). Following the methodology proposed by other PAI factor analytic studies, we first conducted a CFA at the item level, for each individual scale, to test the unidimensionality of the 22 scales. Then, CFAs at the scale level were performed to examine the fit of the factor structure proposed by Morey (1991) for the 11 clinical scales (i.e., two-factor solution obtained in the community standardization sample) and the 22 full scales (i.e., four-factor solution) of the Portuguese version of the PAI. Additionally, we tested other competing factor structures and the three-factor solution for the 11 clinical scales found in Morey’s (1991) clinical standardization sample. To our knowledge, this is the first study that examined several competing factor models through CFA, aiming to find the most parsimonious factor solution of the PAI.

**Results:**

CFAs at the item level suggested an adequate model fit for almost all scales. The results supported a bifactor model with two first-order factors for the 11 clinical scales (the bifactor model with three first-order factor also revealed an adequate fit) and a three-factor model for the 22 full scales. Our findings suggested that each clinical scale is simultaneously accounted for by its specific factor (internalization or externalization factors) and a broad general psychopathology factor.

**Discussion:**

Implications and directions for future research on PAI’s dimensionality are discussed. In a practical manner, the present study may aid practitioners to better understand the psychological functioning of their patients resorting to the PAI. From PAI’s results, technicians can adapt their intervention programs, both in clinical and forensic settings.

## Introduction

Traditionally, psychiatric diagnostic processes have followed the guidelines and models proposed by the Diagnostic and Statistical Manual of Mental Disorders (DSM-5-TR; American Psychiatric Association, 2022) and the International Classification of Diseases (ICD-11; World Health Organization, 2022). These models integrate psychopathology into categories, aiding technicians in carrying out diagnostic processes in a reliable and clear manner (Kendell and Jablensky, 2003). More recently, dimensional models have gained popularity, with special attention being given to the Hierarchical Taxonomy of Psychopathology (HiTOP). This model states that, contrary to traditional conceptualizations, psychopathology is dimensional, has co-occurring features, and it can be organized hierarchically (Kotov et al., 2017, 2021).

Psychopathology can be objectively assessed through robust and valid psychometric measures, one of the most popular being the Personality Assessment Inventory (PAI). The PAI is an objective self-report test of personality, designed to measure psychopathology and personality (Morey, 1991). It contains 344 items, comprising 22 non-overlapping scales: four validity scales, 11 clinical scales, five treatment scales, and 2 interpersonal scales.

Despite the generalized agreement about PAI’s clinical, a substantial amount of discussion persists regarding its factor structure. In the original exploratory factor analysis study, four factors were obtained for the 22 full scales (Morey, 1991). Factor 1 was associated with general severity of symptomatology and impairment in functioning (12 scales: Negative Impression [NIM], Anxiety [ANX], Depression [DEP], Anxiety-Related Disorders [ARD], Schizophrenia [SCZ], Borderline Features [BOR], Somatic Complaints [SOM], Paranoia [PAR], Suicidal Ideation [SUI], Stress [STR], and high negative loadings on Positive Impression [PIM] and Treatment Rejection [RXR]); Factor 2 was defined by behavioral acting-out, impulsivity, and poor judgment (four scales: NIM, Antisocial Features [ANT], Alcohol Problems [ALC], and Drug Problems [DRG]); Factor 3 was defined by egocentricity, exploitativeness, and hostility in interpersonal relationships (four scales: Mania [MAN], Dominance [DOM], ANT, and Aggression [AGG]); and Factor 4 involved social detachment and touchiness and sensitivity in social relationships (six scales: Nonsupport [NON], PAR, SCZ, and high negative loadings on Warmth [WRM] for a community group, and Inconsistency [ICN] and Infrequency [INF] for a clinical group).

For the 11 clinical scales, Morey (1991) conducted two independent exploratory factor analyses, one for the community standardization sample, and another for the clinical standardization sample. Morey found two different factor solutions: (i) a two-factor solution for the community sample (Factor 1 with SOM, ANX, ARD, DEP, PAR, SCZ, and BOR; and Factor 2 with ANT, ALC, and DRG); and (ii) a three-factor solution for the clinical sample (Factor 1 with SOM, ANX, ARD, DEP, PAR, SCZ, and BOR; Factor 2 with ANT, ALC, and DRG; and Factor 3: MAN and ANT).

Mixed findings have been found in the literature for the factor structure of the 22 full scales. While some authors found support for the original four-factor structure (e.g., Burneo-Garcés et al., 2020; Deisinger, 1995; Groves and Engel, 2007), others have suggested a three-factor structure (e.g., Hoelzle and Meyer, 2009; Pignolo et al., 2018; Yoon et al., 2020), or a five-factor solution (e.g., Bach-Nguyen and Morey, 2018; Ortiz-Tallo et al., 2011; Stover et al., 2015).

Some reasons may explain the different factor structures found in the literature, namely sample characteristics (e.g., community vs. clinical samples) and methodology (e.g., number of scales included in the factor analysis, validity criteria, factor analysis techniques [extraction methods: eigenvalues >1 and parallel analysis; EFA, CFA, ESEM], and estimators [ML, MLM, MLR]).

Groves and Engel (2007), for instance, conducted a principal component analysis (PCA) that led them to extract four factors for the 22 full scales of the German version of the PAI, and two factors for the 11 clinical scales. Good to excellent congruence coefficients were found for the four factors and 22 scales between the German and the U.S. samples (i.e., ranging from 0.93 to 0.99), and borderline to excellent congruence coefficients for the two factors and 11 clinical scales (i.e., ranging from 0.89 to 0.99).

Hoelzle and Meyer (2009) extracted three components through a parallel analysis for the 22 scales. The first component described general distress and symptomatology, the second component reflected energetic dominance, inflated self-esteem, stimulus seeking, and aggressiveness, and the third one emphasized externalizing problems, including aggressive impulsivity, rule-breaking, substance abuse, and carelessness (see Table 1). The authors also investigated congruence coefficients across several studies and found that they ranged from 0.76 to 0.99 when Morey’s (1991) sample was the target, and from 0.87 to 0.99 when their sample was the target.

**Table 1:** Rotated pattern component matrix for the 22 PAI scales for the Portuguese, U.S., Italian, and Hoelzle and Meyer (2009) samples.

	Portuguese sample			U.S. normative sample				Italian sample			U.S. sample (Hoelzle and Meyer, 2009)		
	1	2	3	1	2	3	4	1	2	3	1	2	3
Validity scales													
ICN	0.09	−0.14	**0.62**	0.26	**0.63**	−0.09	0.23	0.11	−0.28	**0.59**	0.17	−0.14	**0.55**
INF	0.06	0.02	0.28	0.06	**0.71**	0.01	0.16	−0.05	0.00	0.25	−0.15	−0.24	**0.42**
NIM	**0.79**	0.11	0.15	**0.61**	**0.53**	0.10	0.14	**0.73**	0.02	0.05	**0.78**	−0.02	0.05
PIM	**−0.68**	−0.28	−0.04	**−0.66**	0.21	−0.37	−0.21	**−0.69**	−0.25	0.00	**−0.60**	−0.38	−0.07
Clinical scales													
SOM	**0.60**	−0.00	0.01	**0.65**	**0.43**	−0.09	−0.12	**0.64**	−0.07	−0.20	**0.67**	0.03	−0.18
ANX	**0.88**	−0.00	−07	**0.81**	0.28	−0.02	0.17	**0.87**	−0.09	−0.20	**0.81**	−0.09	−0.08
ARD	**0.82**	0.08	−0.15	**0.79**	0.11	0.05	0.03	**0.82**	0.00	−0.23	**0.83**	0.05	−0.15
DEP	**0.86**	−0.15	0.19	**0.75**	0.37	−0.13	0.29	**0.81**	−0.30	−0.05	**0.91**	−0.18	−03
MAN	0.35	0.75	−11	0.31	0.05	**0.76**	−0.13	**0.43**	**0.70**	−0.01	0.19	**0.77**	0.01
PAR	**0.62**	0.24	0.26	**0.48**	0.36	0.28	**0.46**	**0.66**	0.10	0.13	**0.76**	0.13	−0.08
SCZ	**0.81**	0.05	0.23	**0.62**	0.39	0.12	**0.40**	**0.76**	−0.05	0.14	**0.78**	−0.01	0.18
BOR	**0.85**	0.28	0.14	**0.70**	0.24	0.37	0.34	**0.84**	0.17	0.07	**0.82**	0.21	0.14
ANT	0.14	**0.68**	**0.43**	0.21	**0.51**	**0.60**	0.25	0.23	**0.59**	**0.49**	0.11	**0.54**	**0.60**
ALC	0.02	0.34	**0.48**	0.05	**0.54**	**0.40**	0.25	0.06	0.23	**0.60**	−0.04	0.23	**0.73**
DRG	−0.06	0.21	**0.66**	0.14	**0.78**	0.17	0.06	−0.13	0.00	**0.70**	0.06	0.12	**0.73**
Treatment scales													
AGG	0.33	**0.58**	0.22	0.32	0.22	**0.63**	0.30	**0.45**	**0.43**	0.24	0.34	**0.50**	0.26
SUI	**0.64**	−0.05	0.22	**0.55**	**0.46**	**0.50**	0.18	**0.50**	−0.12	0.17	**0.57**	−0.10	0.13
STR	**0.63**	0.21	0.18	**0.61**	0.12	0.24	0.19	**0.56**	0.18	0.09	**0.66**	0.06	0.00
NON	**0.50**	−0.09	**0.48**	0.36	0.35	0.09	**0.59**	**0.44**	−0.20	0.33	**0.68**	−0.11	0.04
RXR	**−0.77**	−0.04	−0.12	**−0.55**	0.04	−0.19	−07	**−0.73**	0.00	0.03	**−0.71**	−0.11	−0.07
Interpersonal scales													
DOM	−0.35	**0.66**	−0.18	−0.28	−0.13	**0.67**	−0.33	−0.26	**0.71**	−0.07	−0.36	**0.81**	−0.09
WRM	−0.39	0.29	**−0.43**	−0.11	−0.19	0.12	**−0.84**	−0.31	**0.42**	−0.32	**−0.51**	0.30	−0.29
Variance explained (%)	36.9	10.36	7.69	41.4	9.7	8.3	4.7	34.31	9.98	7.84	35.39	9.54	12.19

Components are numbered as in Morey (1991). Loadings ≥ 0.40 are bolded for emphasis.

Pignolo et al. (2018), through a PCA, also extracted three components for the 22 full scales in the Italian version of the PAI. The first component was thought to assess symptomatology and general distress, the second reflected elevated mood and dominance, and the third searched to measure substance abuse and psychopathy (see Table 1). After extracting these three components, the authors computed congruence coefficients to assess similarities between the component structure of the Italian sample and the U.S. sample collected by Hoelzle and Meyer (2009). Results showed good to excellent congruence coefficients between the two studies (e.g., congruence coefficients = 0.99 for Component 1, 0.97 for Component 2, and 0.96 for Component 3, when the Italian sample was selected). The authors did not investigate the factor structure for the 11 clinical scales.

Some studies also analyzed the factor structure for the 11 clinical scales and compared them to Morey’s (1991) two-factor (for the community sample) or three-factor (for the clinical sample) solutions. For example, Cheung et al. (1996) and Groves and Engel (2007) confirmed the original two-factor solution for the 11 clinical scales through a PCA.

Confirmatory factor analysis (CFA) studies with the PAI are limited (some exceptions: Abilleira and Rodicio-García, 2019; Blonigen et al., 2010; Busse et al., 2014; Hopwood and Moser, 2011), and tend to find that Morey’s component structures for the 22 full scales and 11 clinical scales do not meet acceptable fit. For example, Busse et al. (2014) conducted a CFA in a mixed neuropsychological sample to test Morey’s original four-factor model for the 22 full scales and the three-factor model for the 11 clinical scales (i.e., the factor structure found by Morey in its clinical standardization sample). The authors found that these factor models did not reach adequate fit, leading them to conduct a series of PCAs that resulted in a five-component solution for the 22 full scales and a two-component solution for the 11 clinical scales (i.e., the factor structure found in the Morey’s (1991) community standardization sample).

Considering the lack of consensus regarding the PAI internal structure, the current study aimed to investigate the factor structure of the Portuguese version of the PAI and compare it with findings from other languages versions. Following the methodology proposed by other PAI factor analytic studies (e.g., Groves and Engel, 2007; Hoelzle and Meyer, 2009; Morey, 2007; Pignolo et al., 2018), we first conducted a CFA at the item level, for each individual scale, to test the unidimensionality of the 22 scales. Then, CFAs at the scale level were performed to examine the fit of the factor structure proposed by Morey (1991) for the 11 clinical scales (i.e., two-factor solution obtained in the community standardization sample) and the 22 full scales (i.e., four-factor solution) of the Portuguese version of the PAI. Additionally, we tested other competing factor structures (e.g., uncorrelated, correlated, hierarchical, and bifactor models for the 11 clinical scales and the 22 full scales) and the three-factor solution for the 11 clinical scales found in Morey’s (1991) clinical standardization sample. To our knowledge, this is the first study that examined several competing factor models through CFA, aiming to find the most parsimonious factor solution of the PAI.

## Method

### Participants and procedures

The Portuguese standardization sample of the PAI, recruited online, from April 2021 to March 2022, was used in the present study (*N* = 900; 18–74 years, *M* = 43.13, *SD* = 14.28; men, *n* = 381; women, *n* = 519). Participants were recruited from various regions of Portugal (i.e., North, Central, Lisbon, Alentejo, Algarve, Madeira, and Azores) and were selected based on stratified criteria (i.e., gender, age, education, and region), according to the Portuguese Census (PORDATA, 2021). All data was collected online, through *Google Forms*. Descriptive statistics for the sociodemographic characteristics of the Portuguese sample are reported in Table 2. The study was approved by the Faculty of Psychology and Education Sciences of the University of Coimbra Ethics Committee (CEDI/FPCEUC:78/R_5). Participation was voluntary, the objectives of the study were fully explained, and all participants read and signed an informed consent form before taking part in the study. Participants did not receive any kind of compensation for their involvement in the study.

**Table 2:** Sociodemographic characteristics of the Portuguese sample.

	*n*	% of sample
Age (years)		
18–24	108	12.0
25–29	88	9.8
30–39	173	19.2
40–49	217	24.1
50–59	174	19.3
60–74	140	15.6
Gender		
Male	381	42.3
Female	519	57.7
Education level		
Primary education	22	2.4
Middle school	68	7.6
High school	307	34.1
Tertiary education	503	55.9
Region		
North	237	26.3
Center	192	21.3
Lisbon	301	33.4
Alentejo	68	7.6
Algarve	46	5.1
Madeira	31	3.4
Azores	25	2.8
Marital Status		
Single	316	35.1
Married/Registered partnership	463	51.4
Divorced/Separated	113	12.6
Widowed	8	0.9

### Measures

#### Sociodemographic questionnaire

Participants answered a short questionnaire in order to collect personal data on age, gender, education, marital status, and geographic location.

#### Personality Assessment Inventory (PAI)

The PAI is a self-report personality inventory comprised of 344 items, organized into 22 scales (i.e., four validity scales, 11 clinical scales, five treatment consideration scales, and two interpersonal scales), and 10 subscales (Morey, 1991). Items are presented on a 4-point scale (i.e., false, slightly true, mainly true, or very true). The Portuguese version of the PAI was developed and made available by Hogrefe Publishing, which approved the current adaptation and validation study (e.g., translation, back translation, preliminary version, and normative data). The adaptation process followed the International Test Commission guidelines for test adaptation, therefore guaranteeing the quality of its translation and its final version (Hernández et al., 2020).

### Statistical analyses

Raw scores were used in all the statistical analyses. Descriptive statistics, reliability, and PCA were performed using IBM SPSS Statistics 29 (IBM Corp, 2022). To analyze whether the factor structure of the 11 clinical scales and the 22 full scales replicated the original factor structure of the PAI (Morey, 1991), CFAs were conducted using R statistical software (lavaan and semTools packages) at the item and scale level. For interpretation purposes, a CFI > 0.95 (>0.90 may be acceptable for more complex models; Bentler and Bonett, 1980), a RMSEA <0.06, and a SRMR <0.08 were used to determine a good model fit (Hu and Bentler, 1999). For the RMSEA, other cutoff values were also suggested: <0.05 good fit, 0.05–0.08 acceptable fit, 0.08–0.10 mediocre fit, and >0.10 poor fit (Byrne, 2012). Ideally, all three criteria should reach acceptable fits. The Akaike Information Criterion (AIC) was used to compare models, with smaller values representing a better fit.

If CFA did not find evidence for a good fit for the 11 clinical scales and/or for the 22 full scales, we conducted a PCA to find the most parsimonious factor structure following Morey’s (1991) methodology (i.e., PCA with varimax [orthogonal] rotation and congruence coefficients), which was also reproduced by other studies (e.g., Busse et al., 2014; Hoelzle and Meyer, 2009; Pignolo et al., 2018; Tasca et al., 2002; Karlin et al., 2005). Parallel analysis was used to extract the number of components to retain. Congruence coefficients were computed through Barrett’s (2019) Orthosim 3 program. For interpretation purposes, we considered congruence coefficients ranging from 0.98 to 1 as indicative of excellent congruence between dimensions, from 0.92 to 0.98 as good congruence, and from 0.82 to 0.92 as borderline (MacCallum et al., 1999).

## Results

### Descriptive statistics and reliability

Descriptive statistics of the raw scores and reliability (Cronbach alpha) are presented in Table 3. In general, the univariate statistics of skewness and kurtosis yielded adequate values, the exceptions were the NIM, ALC, DRG, and SUI scales. Cronbach alpha ranged from 0.58 (DRG) to 0.92 (ANX) with most scales showing adequate internal consistency (e.g., PAR *α* = 0.87, DEP *α* = 0.89, SUI *α* = 0.91). We did not conduct reliability analysis for ICN and INF (validity scales) because these scales do not measure substantive theoretical constructs and their items were uncorrelated (i.e., ICN scores are the difference between pairs of items; INF shows low-frequency scores in both clinical and non-clinical individuals; in general, studies did not report coefficient alpha for these scales; for a review, see Morey, 2007).

**Table 3:** Descriptive statistics and reliability.

	# of items	*M*	*SD*	Min.	Max.	*α*	Skewness	Kurtosis
Validity scales								
Inconsistency (ICN)	10	5.39	2.38	0	11	—	0.30	−0.42
Infrequency (INF)	8	3.40	2.06	0	8	—	0.21	−0.76
Negative Impression (NIM)	9	2.02	2.50	0	13	0.67	1.78	3.32
Positive Impression (PIM)	9	15.68	3.95	3	22	0.68	−0.60	0.03
Clinical scales								
Somatic Complaints (SOM)	24	13.29	9.32	0	50	0.87	1.14	0.93
Conversion (SOM-C)	8	2.64	3.26	0	21			
Somatization (SOM-S)	8	5.65	3.95	0	18			
Health Concerns (SOM-H)	8	5.00	3.76	0	21			
Anxiety (ANX)	24	23.33	12.16	1	68	0.92	0.91	0.59
Cognitive (ANX-C)	8	8.69	4.69	0	24			
Affective (ANX-A)	8	8.12	4.41	0	24			
Physiological (ANX-P)	8	6.52	4.13	0	23			
Anxiety-Related Disorders (ARD)	24	21.89	9.60	2	65	0.83	0.96	1.12
Obsessive-Compulsive (ARD-O)	8	9.03	3.86	0	24			
Phobias (ARD-P)	8	7.62	3.98	0	21			
Traumatic Stress (ARD-T)	8	5.24	4.87	0	24			
Depression (DEP)	24	19.65	10.70	0	60	0.89	1.03	1.01
Cognitive (DEP-C)	8	6.24	3.78	0	23			
Affective (DEP-A)	8	5.74	4.40	0	23			
Physiological (DEP-P)	8	7.67	4.43	0	21			
Mania (MAN)	24	21.23	8.65	1	55	0.82	0.62	0.53
Activity Level (MAN-A)	8	6.42	3.12	0	20			
Grandiosity (MAN-G)	8	6.38	3.82	0	22			
Irritability (MAN-I)	8	8.43	4.24	0	24			
Paranoia (PAR)	24	24.51	9.69	4	64	0.87	0.66	0.53
Hypervigilance (PAR-H)	8	10.94	3.86	1	24			
Persecution (PAR-P)	8	4.37	3.74	0	21			
Resentment (PAR-R)	8	9.19	3.88	0	24			
Schizophrenia (SCZ)	24	15.96	8.29	0	52	0.84	0.94	0.98
Psychotic Experiences (SCZ-P)	8	3.70	2.97	0	15			
Social Detachment (SCZ-S)	8	6.97	4.26	0	22			
Thought Disorder (SCZ-T)	8	5.30	3.82	0	22			
Borderline Features (BOR)	24	23.09	10.39	4	60	0.87	0.69	0.13
Affective Instability (BOR-A)	6	6.22	3.21	0	18			
Identity Problems (BOR-I)	6	6.17	3.57	0	18			
Negative Relationships (BOR-N)	6	6.69	3.41	0	17			
Self-Harm (BOR-S)	6	3.99	2.98	0	15			
Antisocial Features (ANT)	24	12.83	6.67	1	43	0.73	1.01	1.34
Antisocial Behaviors (ANT-A)	8	4.11	3.57	0	20			
Egocentricity (ANT-E)	8	2.73	2.26	0	13			
Stimulus-Seeking (ANT-S)	8	5.98	2.91	0	19			
Alcohol Problems (ALC)	12	3.08	3.79	0	25	0.75	2.21	6.69
Drug Problems (DRG)	12	2.81	3.41	0	26	0.58	2.16	7.75
Treatment scales								
Aggression (AGG)	18	14.22	7.14	0	43	0.82	0.65	0.31
Aggressive Attitude (AGG-A)	6	6.22	3.10	0	16			
Verbal Aggression (AGG-V)	6	6.18	3.22	0	15			
Physical Aggression (AGG-P)	6	1.82	2.23	0	14			
Suicidal Ideation (SUI)	12	3.50	5.74	0	36	0.91	2.59	7.43
Stress (STR)	8	6.96	3.66	0	23	0.63	0.84	1.05
Nonsupport (NON)	8	7.04	3.99	0	20	0.75	0.41	−0.36
Treatment Rejection (RXR)	8	15.21	4.21	1	24	0.77	−0.59	0.11
Interpersonal scales								
Dominance (DOM)	12	20.18	5.15	4	35	0.78	−0.10	−0.06
Warmth (WRM)	12	20.97	5.57	3	35	0.82	−0.10	−0.27

### Preliminary analysis

**Figure 1:**
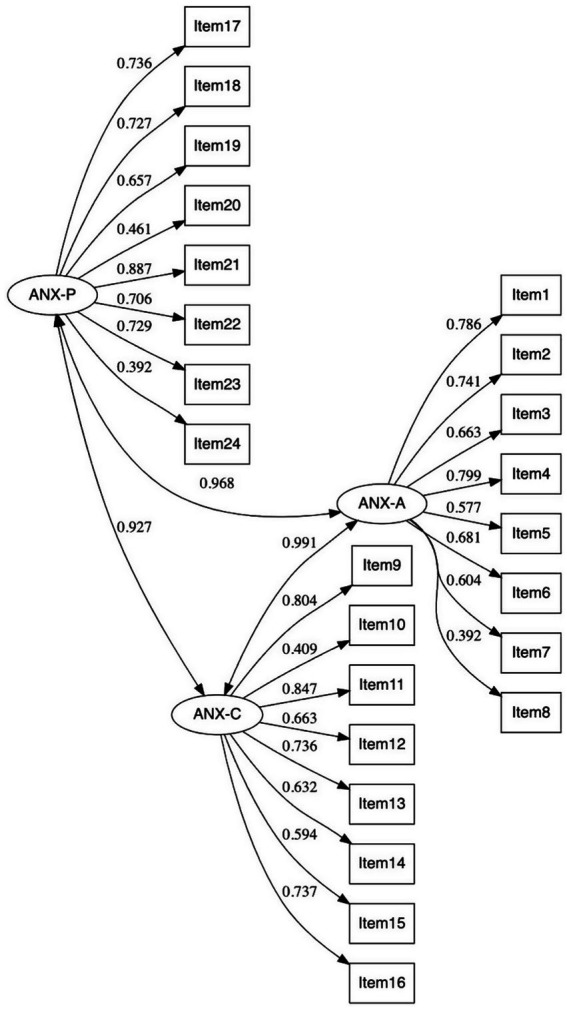
CFA of ANX scale at the item level (standardized solution). Three latent variables (i.e., one for each ANX subscale) and 24 observed variables (i.e., items). Reliability for ANX-P: McDonald’s *ω* = 0.798; ANX-A: McDonald’s *ω* = 0.808; and ANX-C: McDonald’s *ω* = 0.824.

To perform CFAs at the scale level (i.e., scales were treated as continuous indicators), we first analyzed the fit of the 22 PAI scales at the item level (i.e., individual PAI items were considered as observed variables). Figure 1 illustrates the factor model for the ANX scale. The Weighted Least Squares Mean, and Variance adjusted (WLSMV) estimator was used because indicators were ordinal variables (WLSMV is robust to violations of multivariate normality and uses a polychoric correlation matrix). As shown in Table 4, the goodness-of-fit indices at the item level were adequate for most scales (e.g., RXR scale: CFI = 0.98, RMSEA = 0.06, SRMR = 0.04; NIM scale: CFI = 0.98, RMSEA = 0.03, SRMR = 0.06).

**Table 4:** Goodness-of-fit indices of PAI scales (item level).

	χ^2^	CFI	RMSEA (90%CI)	SRMR
Validity scales				
Inconsistency (ICN)	—	—	—	—
Infrequency (INF)	—	—	—	—
Negative Impression (NIM)	48.578	0.982	0.030 (0.016–0.043)	0.056
Positive Impression (PIM)	171.213	0.902	0.077 (0.066–0.088)	0.063
Clinical scales				
Somatic Complaints (SOM)	1,407.035	0.942	0.072 (0.069–0.076)	0.090
Anxiety (ANX)	1,288.686	0.944	0.068 (0.064–0.072)	0.056
Anxiety-Related Disorders (ARD)	1,409.860	0.920	0.072 (0.069–0.076)	0.075
Depression (DEP)	1,419.777	0.938	0.073 (0.069–0.077)	0.064
Mania (MAN)	923.470	0.899	0.055 (0.051–0.059)	0.064
Paranoia (PAR)	1,420.452	0.901	0.073 (0.069–0.077)	0.071
Schizophrenia (SCZ)	1,258.563	0.905	0.068 (0.064–0.071)	0.089
Borderline Features (BOR)	1,332.329	0.906	0.010 (0.067–0.074)	0.071
Antisocial Features (ANT)	730.426	0.887	0.047 (0.043–0.051)	0.081
Alcohol Problems (ALC)	331.310	0.961	0.087 (0.079–0.096)	0.101
Drug Problems (DRG)	117.178	0.965	0.043 (0.034–0.053)	0.102
Treatment scales				
Aggression (AGG)	596.068	0.919	0.063 (0.058–0.068)	0.068
Suicidal Ideation (SUI)	351.365	0.988	0.088 (0.089–0.097)	0.058
Stress (STR)	73.748	0.956	0.059 (0.045–0.073)	0.049
Nonsupport (NON)	105.004	0.979	0.076 (0.062–0.090)	0.054
Treatment Rejection (RXR)	69.681	0.980	0.056 (0.043–0.071)	0.035
Interpersonal scales				
Dominance (DOM)	440.767	0.912	0.095 (0.087–0.104)	0.073
Warmth (WRM)	391.180	0.942	0.087 (0.079–0.095)	0.064

Fit indices for ICN (scores are the difference between pairs of items) and INF (low frequency in normal and clinical individuals) were not included given the nature of these scales. *χ*^2^ = chi-square. CFI = comparative fit index. RMSEA (90% CI) = root mean square error of approximation (90% confidence interval). SRMR = standardized root mean square residual.

### CFA for the 11 clinical scales

Following Morey’s (1991, 2007) factor structure for the 11 clinical scales, we tested eight competing factor structures with two- and three-factors; and uncorrelated (to analyze if the clinical scales operate independently), correlated (to analyze if the clinical scales share some underlying personality processes), hierarchical (to analyze if the clinical scales could be explained by a higher-order factor of personality), and bifactor models (to analyze the presence of a broad general personality factor). The eight competing factor structures tested were: (i) a two-uncorrelated-factor model with the SOM, ANX, ARD, DEP, PAR, SCZ, and BOR in the first factor and the MAN, ANT, ALC, and DRG in the second factor; (ii) a two-correlated-factor model; (iii) a hierarchical two-factor model with a general factor (second-order factor), the two first-order factors, and the 11 clinical scales; (iv) a three-uncorrelated-factor model with the SOM, ANX, ARD, DEP, PAR, SCZ, and BOR in the first factor, the ANT, ALC, and DRG in the second factor, and MAN and ANT in the third factor; (v) a three-correlated-factor model; (vi) a hierarchical three-factor model with a general factor (second-order factor), the three first-order factors, and the 11 clinical scales; (vii and viii) a bifactor model where each clinical scale loads onto its respective first-order factor (two or three first-order factors) and simultaneously onto a general factor. For the CFA at the scale level, we used the maximum likelihood parameter with standard errors and a mean-adjusted chi-square test statistic estimator (MLM; also referred to as the Satorra-Bentler chi-square) that is robust to non-normality (Mardia’s multivariate normality test for the 11 clinical scales: kurtosis = 183.715, *p* < 0.001; skewness = 19.862, *p* < 0.001).

A preliminary analysis of the modification indices for each of the factor models suggested the addition of five error covariances (ARD – ANX, MAN – DEP, PAR – ANX, MAN – ARD, and DRG – MAN), which were accordingly added for all models. The addition of these error covariance makes empirical and statistical sense because these scales may share some underlying personality constructs and are significantly correlated (e.g., ARD – ANX *r* = 0.793, PAR – ANX *r* = 0.456).

Goodness-of-fit indices are presented in Table 5. The bifactor models for the two and three first-order factors showed a better fit than the other competing factor models, with most of the scales showing higher loadings in their respective first-order factors than in the general factor (see Table 6). However, the bifactor model with two first-order factors seems to be a more parsimonious factor structure because it did not include a cross-loading, and the number of indicators (or observed variables) per factor was more adequate (the bifactor model with three first-order factors had a factor with only two indicators). The hierarchical three-factor model was inadmissible (Heywood case).

**Table 5:** Goodness-of-fit indices of the PAI scales (scale level).

	*χ* ^2^	*df*	CFI	RMSEA (90%CI)	SRMR	AIC
11 clinical scales						
2-uncorrelated-factor	468.756	39	0.900	0.122 (0.112–0.132)	0.137	64,290.025
2-correlated-factor	393.200	38	0.918	0.113 (0.102–0.122)	0.079	64,196.658
Hierarchical 2-factor	527.181	39	0.885	0.131 (0.121–0.141)	0.131	64,367.601
Bifactor model (2 factors)	164.794	28	0.970	0.079 (0.068–0.091)	0.041	63,934.162
3-uncorrelated-factor	464.977	39	0.901	0.121 (0.111–0.131)	0.138	64,283.157
3-correlated-factor	379.100	36	0.922	0.112 (0.102–0.123)	0.080	64,178.549
Hierarchical 3-factor	Heywood case					
Bifactor model (3 factors)	159.398	28	0.971	0.078 (0.066–0.090)	0.037	63,928.653
22 full scales						
4-uncorrelated-factor	Heywood case					
4-correlated-factor	1,760.342	195	0.827	0.103 (0.099–0.108)	0.092	115,266.006
Hierarchical 4-factor	1,814.007	197	0.821	0.104 (0.100–0.109)	0.100	115,327.503
Bifactor model (4 factors)	1,447.927	180	0.860	0.097 (0.092–0.101)	0.066	114,922.932

*χ*^2^ = chi-square. df = degrees of freedom. CFI = comparative fit index. SRMR = standardized root mean square residual. RMSEA (90% CI) = root mean square error of approximation (90% confidence interval). AIC = Akaike information criterion.

**Table 6:** Loadings of the bifactor model for the 11 clinical scales.

	Bifactor with two first-order factors				Bifactor with three first-order factors				
	F1	F2	GF	*R^2^*	F1	F2	F3	GF	*R^2^*
SOM	0.572		0.152	0.350	0.600			0.151	0.383
ANX	0.858		0.233	0.790	0.810			0.338	0.771
ARD	0.743		0.228	0.604	0.668			0.363	0.578
DEP	0.816		0.245	0.725	0.815			0.305	0.757
PAR	0.509		0.462	0.472	0.411			0.573	0.497
SCZ	0.678		0.437	0.650	0.613			0.527	0.653
BOR	0.709		0.528	0.782	0.624			0.641	0.800
MAN		0.171	0.737	0.572			−0.333	0.635	0.514
ANT		0.288	0.744	0.637		0.440	−0.431	0.576	0.711
ALC		0.404	0.306	0.257		0.461		0.247	0.273
DRG		0.545	0.237	0.353		0.536		0.173	0.317
*ω*	0.90	0.18	0.80		0.89	0.58	0.40	0.83	
ECV			0.34					0.35	

GF = general factor. *R*^2^ = communalities (variance explained by the latent factors). *ω* = McDonald’s *ω*. ECV = explained common variance (i.e., the general factor explains 34 and 35% of the common variance extracted; see Reise et al., 2013; Rodriguez et al., 2016).

### CFA and PCA for the 22 full scales

Although the literature proposed other factor structures for the 22 full scales (three-, four-, and five-factor models), we only analyzed Morey’s (1991, 2007) original four-factor model. The study of other factor structures identified by the literature for the 22 full scales was out of the scope of the present study because mixed findings have been found even in the studies that found the same number of latent factors (e.g., different number of scales included in each of the latent factors; Hoelzle and Meyer, 2009; Pignolo et al., 2018). Similarly to the previous CFA for the clinical scales, we tested four competing factor structures for the 22 full scales: (i) a four-uncorrelated-factor model (Factor 1: NIM, PIM, SOM, ANX, ARD, DEP, PAR, SCZ, BOR, SUI, STR, and RXR; Factor 2: INC, INF, ALC, and DRG; Factor 3: MAN, ANT, AGG, and DOM; Factor 4: NON and WRM); (ii) a four-correlated-factor model; (iii) a hierarchical four-factor model with a general factor (second-order factor), the four first-order factors, and the 22 scales; and (iv) a bifactor model where each scale loads onto its respective first-order factor and simultaneously onto a general factor. The MLM was used as estimator (Mardia’s multivariate normality test for the 22 full scales: kurtosis = 634.852, *p* < 0.001; skewness = 50.266, *p* < 0.001). Modification indices for each of the factor models suggested the addition of eight error covariances. As shown in Table 5, we found a poor fit for three models and an inadmissible factor structure for the four-uncorrelated-factor model.

Since the CFA did not provide evidence for a good fit for the 22 full scales, a PCA was conducted to find the most parsimonious factor structure. The same approach was used by Busse et al. (2014) in a mixed neuropsychological sample. Following Morey’s original methodology, PCA with varimax (orthogonal) rotation and congruence coefficients analyses were performed on the 22 full scales. Parallel analysis suggested three components with eigenvalues exceeding the corresponding criterion values for a randomly generated data matrix of the same size (i.e., 22 variables and 900 participants). The three components explained a total of 55.05% of the variance (Kaiser-Meyer-Olkin = 0.912, Bartlett’s test of sphericity with *p* < 0.001).

Table 1 shows the three-factor solution found in the Portuguese version and in three international versions [i.e., U.S. normative sample, Italian sample, and Hoelzle and Meyer’s (2009) U.S. sample] that also conducted a PCA with varimax rotation. The first component (36.99% of variance) was characterized by higher loadings in the NIM, PIM, SOM, ANX, ARD, DEP, PAR, SCZ, BOR, SUI, STR, NON, and RXR scales. The second component (10.36% of variance) showed higher loadings in the MAN, ANT, AGG, and DOM scales, whereas the third component (7.69% of variance) in the ICN, ANT, ALC, DRG, NON, and WRM scales. Thus, the first component can be described as measuring symptomatology and general distress, the second component as assessing elevated mood and dominance, and the third component as reflecting substance abuse and psychopathy.

These findings are consistent with those reported in the Italian adaptation of the PAI (Pignolo et al., 2018) and other studies (e.g., Hoelzle and Meyer, 2009) that also supported the three-factor structure of the PAI.

Additionally, we computed congruence coefficients between the three-factor solution found in our Portuguese sample and the Pignolo et al. (2018; Italian sample) and Hoelzle and Meyer (2009; USA sample) studies. The congruence coefficients (row normalization) were 0.98 for Component 1, 0.99 for Component 2, and 0.99 for Component 3 when the Portuguese sample was selected as the target matrix (0.99, 0.99, and 0.99 when the Italian sample was selected as the target matrix, respectively). To the factor solution obtained by Hoelzle and Meyer (2009), congruence coefficients were 0.98, 0.96, and 0.95 when the Portuguese sample was selected as the target matrix (0.98, 0.96, and 0.94 when Hoelzle and Meyer’s sample was selected as the target matrix, respectively). These results suggested good to excellent congruence between PAI’s Portuguese version and either the USA or the Italian versions.

## Discussion and conclusions

According to the American Educational Research Association et al., (2014) *Standards for Educational and Psychological Testing*, there are five sources of validity evidence that can be used to assess the validity of a given test, namely (i) test content, (ii), response processes, (iii) internal structure, (iv) relations to other variables, and (v) testing consequences (American Educational Research Association et al., 2014). Given that these sources of validity have been exhaustively studied for the PAI in past research (including in the original PAI manual; Morey, 1991), the present study serves to further contribute to the validation of the PAI by providing evidence of its internal structure, through exploratory and confirmatory factor analysis, two common procedures for assessing the dimensional structure of measures (Sireci and Benítez Baena, 2023).

The factor structure of the PAI has been investigated by several studies, leading to mixed results when it comes to the number of factors extracted or confirmed for the 22 full scales and 11 clinical scales. The present study aimed to investigate the factor structure of the PAI’s Portuguese version, contributing to the existing discussion regarding its dimensionality.

CFAs at the item level suggested an adequate model fit for almost all scales. The goodness-of-fit indices of the Portuguese version were slightly higher than those found for the Italian version (Pignolo et al., 2018), albeit smaller than the ones of the U.S.A. version (Morey, 2007). Thus, globally, the conceptual scale structure (i.e., items and subscales) proposed by Morey (1991, 2007) was confirmed in the Portuguese study.

To our knowledge, the current study is among the most comprehensive about PAI’s dimensionality, considering we compared eight factor models for the 11 clinical scales and four factor models for the 22 full scales through CFA at the scale level. In the 11 clinical scales, we contrasted the two- and the three-factor solutions found by Morey (1991, 2007) in its community and clinical samples, respectively. The bifactor models with two and three first-order factors showed a better fit than the other competing factor models, offering additional support to the factor structure found by Morey (1991). Even though both bifactor models (two and three first-order factors) showed very similar goodness-of-fit indices, the bifactor model with two first-order factors seems to be the most parsimonious factor structure for PAI’s Portuguese version. The bifactor model with three first-order factors had a factor with only two indicators (factor 3: MAN and ANT), and the ANT scale loaded into two factors (i.e., cross-loading). The two first-order factors can be conceptualized as measuring internalization (factor 1) and externalization (factor 2).

Although our findings were somewhat consistent with the two-factor model found in German (Groves and Engel, 2007), Chinese (Cheung et al., 1996), and U.S. (Busse et al., 2014; Morey, 1991, 2007) samples, this was the first study investigating the existence of a general factor by presenting a bifactor solution for PAI’s 11 clinical scales. Our findings suggested that each clinical scale is simultaneously accounted for by its specific factor (internalization or externalization factors) and a broad general psychopathology factor. This proposal is consistent with the current knowledge about the bifactor structure of psychiatric disorders, in which a general psychopathology factor captures what is common across all forms of psychiatric diagnoses and accounts for the co-occurrence of internalizing and externalizing disorders (Gluschkoff et al., 2019). Nevertheless, it is worth noting that an alternative three-factor structure has also been found in other studies both in community (Deisinger, 1995; Stover et al., 2015) and forensic samples (Burneo-Garcés et al., 2020).

For the 22 full scales, we also compared four factor models through CFA, but a poor fit was found for three of these models and an inadmissible structure was observed for the four-uncorrelated-factor model. Thus, a PCA (varimax rotation) was conducted for the 22 full scales, suggesting a three-factor structure (parallel analysis). The three components can be conceptualized as measuring: (i) symptomatology and general distress, (ii) elevated mood and dominance, and (iii) substance abuse and psychopathy. Although this factor structure differs from the four-component solution proposed by Morey (1991) and confirmed in the German (Groves and Engel, 2007), Chinese (Cheung et al., 1996), Greek (Lyrakos, 2011), and Spanish (Burneo-Garcés et al., 2020) studies, it is similar to the factor structure found in the Italian (Pignolo et al., 2018), South-Korean (Yoon et al., 2020), and U.S. (Hoelzle and Meyer, 2009) studies. This factor structure is aligned with the HiTOP model, by differentiating an elevated mood and dominance component from a substance use and psychopathy component. Indeed, differences between these two components may be better explained by the disinhibited externalizing spectra of HiTOP (which includes a substance abuse and antisocial behavior subfactor) and the antagonistic externalizing spectra of HiTOP (see Kotov et al., 2017, 2021). Therefore, our findings are in line with the framework that has significantly contributed to the understanding of the structure of psychopathology.

Congruence coefficients between the Portuguese version, the Italian version (Pignolo et al., 2018), and the factor structure found by Hoelzle and Meyer (2009) for the 22 full scales were found to vary between good and excellent. Congruence coefficients in the current study, ranging from 0.98 to 0.99, were overall higher than the ones obtained by Pignolo et al. (2018) (ranging from 0.92 to 0.97, when compared to Morey’s (1991) and Hoelzle and Meyer’s (2009) sample), and Hoelzle and Meyer (2009) (ranging from 0.76 to 0.99, when compared to Morey’s (1991) sample). These findings seem to suggest that the three-factor solution that was found for the 22 full scales closely resembled the structure obtained by Pignolo et al. (2018) and Hoelzle and Meyer (2009).

Factors such as sample characteristics (e.g., community or clinical) and employed methodology (e.g., number of scales included in the model, validity criteria, extraction methods) may explain the different factor structures found in literature. For example, it can be hypothesized that in the original U.S. version (as in other studies that replicated its methodology) an over-extraction of factors may have occurred due to the extraction methods used (i.e., eigenvalues >1 and the Kaiser criterion). These extraction methods are commonly described as less accurate, resulting in an over-retention of factors (Costello and Osborne, 2005). A possible solution to this over-extraction may be the inclusion of more accurate extraction methods (e.g., parallel analysis), which were used in the current study.

Despite the strengths of the present study (e.g., testing various factor models, reaching excellent congruence with similar international factor structures), some limitations should be considered. First, only community samples were considered. Because the type of sample can influence the dimensionality of a scale, the PAI’s structure, as found in the current study, can only account for non-clinical samples. Future research should include clinical (e.g., psychiatric patients and inpatients) or forensic samples (e.g., victims, offenders, and forensic professionals), considering that the nature of the samples may influence the factor structure of the clinical scales (e.g., the factor structure found by Morey in the clinical sample). Second, it would be necessary to explore the equivalence (measurement invariance) of the factor structure of the PAI across community and clinical samples, and across international versions.

In conclusion, the present study provides evidence regarding the adequate psychometric properties of the PAI, in which the two-factor (bifactor) model for the 11 clinical scales (CFA) and the three-factor model for the 22 full scales (PCA) were the best, and also the most parsimonious, factor structures. Our findings contribute to answer one of the greatest sources of debate on the PAI, particularly its dimensionality. Besides confirming a three-factor model, reinforcing past findings, it also tested new alternative models (e.g., correlated, hierarchical), excluding them. In a practical manner, the present study may aid practitioners to better understand the psychological functioning of their patients resorting to the PAI. For example, if a patient scored highly on ANT, ALC, and DRG scales, clinicians may hypothesize that he/she may have externalization problems. From PAI’s results, technicians can adapt their intervention programs, both in clinical and forensic settings. It is important to continue studying the PAI factor structure, including clinical and forensic samples.

## Data Availability

The datasets presented in this article are not readily available because of ethical and commercial rights (Hogrefe Publisher, Portugal). Requests to access the datasets should be directed to Hogrefe Publisher (Portugal; testes@hogrefe.pt).
